# Atrial and Ventricular Structural and Functional Alterations in Obese Children

**DOI:** 10.3390/medicina57060562

**Published:** 2021-06-02

**Authors:** Rima Šileikienė, Karolina Adamonytė, Aristida Ziutelienė, Eglė Ramanauskienė, Jolanta Justina Vaškelytė

**Affiliations:** 1 Department of Pediatric Cardiology, Medical Academy, Lithuanian University of Health Sciences, Eivenių 2, LT 50009 Kaunas, Lithuania; aristida.ziuteliene@kaunoklinikos.lt; 2 Department of Children Diseases, Medical Academy, Lithuanian University of Health Sciences, LT 47130 Kaunas, Lithuania; karolina.adamonyte27@gmail.com (K.A.); egle.ramanauskiene@kaunoklinikos.lt (E.R.); 3 Department of Cardiology, Medical Academy, Lithuanian University of Health Sciences, LT 44307 Kaunas, Lithuania; jolanta.vaskelyte@kaunoklinikos.lt

**Keywords:** obesity, children, two-dimensional strain, cardiac dysfunction

## Abstract

*Background and objectives*: Childhood obesity has reached epidemic levels in the world. Obesity in children is defined as a body mass index (BMI) equal to or above the 95th percentile for age and sex. The aim of this study was to determine early changes in cardiac structure and function in obese children by comparing them with their nonobese peers, using echocardiography methods. *Materials and methods*: The study enrolled 35 obese and 37 age-matched nonobese children. Standardized 2-dimensional (2D), pulsed wave tissue Doppler, and 2D speckle tracking echocardiography were performed. The z-score BMI and lipid metabolism were assessed in all children. *Results*: Obese children (aged 13.51 ± 2.15 years; 20 boys; BMI z-score of 0.88 ± 0.63) were characterized by enlarged ventricular and atrial volumes, a thicker left ventricular posterior wall, and increased left ventricular mass. Decreased LV and RV systolic and diastolic function was found in obese children. Atrial peak negative (contraction) strain (−2.05% ± 2.17% vs. −4.87% ± 2.97%, *p* < 0.001), LV and RV global longitudinal strain (−13.3% ± 2.88% vs. −16.87% ± 3.39%; −12.51% ± 10.09% vs. −21.51% ± 7.42%, *p* < 0.001), and LV global circumferential strain (−17.0 ± 2.7% vs. −19.5 ± 2.9%, *p* < 0.001) were reduced in obese children. LV torsion (17.94° ± 2.07° vs. 12.45° ± 3.94°, *p* < 0.001) and normalized torsion (2.49 ± 0.4°/cm vs. 1.86 ± 0.61°/cm, *p* = 0.001) were greater in obese than nonobese children. A significant inverse correlation was found between LV and RV global longitudinal strain and BMI (*r* = −0.526, *p* < 0.01; *r* = −0.434, *p* < 0.01) and total cholesterol (*r* = −0.417, *p* < 0.01). Multivariate analysis revealed that the BMI z-score was independently related to LV and RV global longitudinal strain as well as LV circumferential and radial strain. *Conclusion:* 2D speckle tracking echocardiography is beneficial in the early detection of regional LV systolic and diastolic dysfunctions, with preserved ejection fraction as well as additional RV and atrial involvement, in obese children. Obesity may negatively influence atrial and ventricular function, as measured by 2D speckle tracking echocardiography. Obese children, though they are apparently healthy, may have subclinical myocardial dysfunction.

## 1. Introduction

Childhood obesity represents one of the most serious global public health challenges of the 21st century. In the WHO European Region, every third child is overweight or obese [1].

Obesity in children is defined as a body mass index (BMI) equal to or above the 95th percentile for age and sex [2]. In adults, obesity is a significant risk factor for coronary artery disease and congestive heart failure, and is associated with increased morbidity and mortality. The somatic consequences of child obesity are rarely expressed clinically, but lead to serious cardiovascular complications in adulthood [3,4,5]. Conventional echocardiography provides information about the structure and function of the left ventricle (LV). However, conventional echocardiography is not sensitive enough to detect early cardiovascular changes in obese children. Many studies have shown that LV ejection fraction and fractional shortening do not differ between obese and nonobese children [4,6]; however, more sensitive methods such as 2D and 3D speckle tracking imaging have revealed the reduction of systolic myocardial function involving the left and the right ventricle (RV).

The aim of our study was to assess atrial and ventricular systolic and diastolic function in obese children using 2D speckle tracking imaging, to correlate the values with traditional 2D echocardiographic measurements of atrial and ventricular function. We investigated the relationship between the variables of atrial and ventricular deformation and BMI, blood pressure, and lipid profile in obese and nonobese children.

## 2. Materials and Methods

### 2.1. Study Population

Of the 39 obese children without cardiovascular pathology admitted to the Department of Children’s Diseases, Lithuanian University of Health Sciences, between December 2016 and June 2018, four were excluded due to a poor acoustic window or having no suitable view for speckle tracking analysis. Thus, the study group consisted of 35 nonsyndromic obese children (mean age 13.51 ± 2.14, 20 boys). Children in the study group had no history of smoking or clinical signs of cardiac disease, chronic illness, sleep apnea syndrome, diabetes mellitus, or hypertension. Their physical activity was normal (6–7 h/week). Their physical activity was evaluated by a survey of patients and their parents.

Data of the children in the study group were compared with the data of 37 age-matched healthy children (control group); they had no medical complaints, no history of cardiac rhythm disturbance, they did not use any medications, and echocardiography was performed for a heart murmur. The study included children with normal findings of echocardiography. 

Children were weighed in light clothes, using a Seca scale 711 (Medical Measuring Systems and Scales, Germany) with a capacity of 220 kg and accuracy of 100 g. Height was measured with a stadiometer (accuracy of 0.5 cm). The body surface area (BSA) was calculated according to the Dubois formula; BMI was calculated according to the formula: BMI = weight (kg)/height (m^2^). Obesity was defined as when BMI, adjusted to the sex and age, exceeded the 95th percentile [2,7]. Obese and control group children were prospectively enrolled. Systolic blood pressure (SBP) and diastolic blood pressure (DBP) were measured at rest after 10 min by the auscultation method. Measurements of anthropometric characteristics, heart rate, and arterial blood pressure were performed. A 12 h fasting lipid profile was prospectively enrolled in the study, and total cholesterol, low-density lipoproteins (LDLP), high-density lipoproteins (HDLP), and triglycerides were analyzed in obese and nonobese children; according to the Tanner puberty standardized classification, children of both groups and genders matched the III puberty stage. 

### 2.2. Ethics, Consent and Permissions

The study was conducted in accordance with the Declaration of Helsinki; the Bioethics Committee of the Lithuanian University of Health Sciences gave their permission to conduct the study (BEC-MF92, November 2016). Children and their parents gave their informed consent to perform a lipid profile. 

### 2.3. Conventional Echocardiography

2D echocardiography was performed using a commercial ultrasound system (Vivid 7, GE Vingmed Ultrasound AS, Horten, Norway) with a 3.5-MHz transducer. A 3-lead electrocardiogram was taken throughout the examination. All images were recorded using harmonic imaging and stored digitally for analysis. The mean frame rate was 50 frames per second (range of 40 to 70 frames/s). Echocardiography and calculations of morphometric parameters were performed in accordance with the recommendations by the American Society of Echocardiography [8]. 

The biplane Simpson’s method was used for the calculation of LV ejection fraction. 

LV and left atrial (LA) volumes were determined using the modified Simpson’s rule with images obtained from apical four-chamber and two-chamber views. Right atrial (RA) volume was obtained from an apical four-chamber view. A volumetric atrial assessment was performed at the end of systole, just before mitral valve opening, to assess maximal atrial volume, and at the end of diastole, after mitral valve closing, to assess minimal atrial volume. From these parameters, the total stroke volume (maximal atrial volume minus minimal atrial volume) and total emptying fraction (total stroke volume divided by maximal volume × 100) were calculated [9]. 

BMI, LV volumes, and LV dimensions z-scores were calculated based on the normal values of M-mode measurements [10,11]. The amplitude of tricuspid valve annulus excursion was evaluated using an M mode from an apical four-chamber view at the lateral RV wall.

LV mass and atrial volumes were also indexed to body height raised to a power of 2.7.

### 2.4. Doppler Imaging

The diastolic function of LV and RV was assessed using pulsed wave Doppler. The peak velocities of mitral and tricuspid early filling (E) as well as late filling (A) waves, E/A ratio, and mitral E-wave deceleration time were measured. 

From the standard four-chamber view, the function of the LV and RV long axis was assessed using tissue Doppler imaging. The peak systolic velocity of mitral and tricuspid annulus (Sm, St), early diastolic velocity (Em, Et) and late diastolic velocity (Am, At) were recorded and averaged over three consecutive cardiac cycles. The ratio of E/Em was calculated.

### 2.5. 2D Speckle Tracking Imaging

The images obtained by conventional echocardiography were stored on the hard disc of the echocardiographic machine and transferred to a workstation (EchoPac software, GE Healthcare, Milwaukee, WI, USA) for offline analysis. A line was traced along the endocardium of LV and RV at the frame where it was best defined. The LV was divided into 6 long-axis segments in each view (apical four-, three- and two-chamber). The RV lateral wall was divided into basal, middle, and apical segments. For better visualization of the lateral wall of the RV and LV, children were asked to hold their breath. Radial and circumferential strain analysis was performed at the mitral valve, papillary muscle, and apical level in the parasternal short-axis view. 

For atrial analysis, a line was drawn along LA and RA endocardium, when the atria were at their minimal volume after contraction. The RA was divided into 2 (annular and middle) long-axis segments of the lateral wall, and the LA was divided into 4 long-axis segments (annular and middle) of the septum, lateral, anterior, and inferior walls. The superior or “roof” region of the atria was excluded, as this segment is rather stationary and makes no contribution to the atrial motion and active contraction. 

Three cardiac cycles were averaged. For the assessment of LV and RV long-axis function, mean global strain values were calculated. Closure of the aortic valve was identified as a sign of the end-systole. 

LA and RA long-axis function was assessed during atrial contraction (time from the end of P-wave on ECG to mitral valve closure), corresponding to atrial contractile function (peak negative strain), and during atrial relaxation (time from mitral valve closure to aortic valve opening), corresponding to atrial conduit function (peak positive strain). The sum of peak positive and negative strains was considered to be the total atrial strain, corresponding to atrial reservoir function [12]. 

For the assessment of LV short-axis function, the images were scanned at the basis of the heart (mitral valve, papillary muscle, and apical levels). The following parameters were calculated: global radial and circumferential strain, rotation of anterior septum, anterior, lateral, inferior, posterior LV walls and inferior LV septum. Data were subdivided into 6 segments for regional analysis at each ventricular level and averaged at the basal and apical level for the analysis of global rotational motion. Torsion (difference between the basal and apical rotational movements of the heart) and normalized torsion (difference between the basal and apical rotational movements of the heart, divided by the length of the long axis of the LV) [13] were also assessed.

### 2.6. Reproducibility

Intraobserver measurements for ventricular and atrial strains were determined repeating the measurements of 2D speckle tracking echocardiography in 5 randomly selected subjects 1 month later. Intraobserver reproducibility was evaluated by means of the intraclass correlation coefficient (ICC). Differences were considered statistically significant when a *p*-value of <0.05 was obtained. The significance of inter-technique biases was tested using the paired *t*-test. 

### 2.7. Statistical Analysis

The data were processed and analyzed using the IBM SPSS Statistics 22 package. 

The Kolmogorov–Smirnov test was used in the investigation of hypotheses about the normality of the parameter distribution. Descriptive statistical data were presented as mean ± standard deviation (SD), median (interquartile range), number (*n*) and percentage (%). Student’s t-test was used to compare normally distributed data, and the Mann–Whitney test to compare non-normally distributed data. 

The sample size was calculated using the formula *n* = 1/(∆^2^ + 1/N), where *n* is the sample size, ∆, the type error (0.05), and N, the population size. The calculated sample size was 35 children. We calculated the power of our study to substantiate the sample volume (correct finding of the difference between the groups, reasonably rejecting the null (H0) hypothesis when the type I error α = 0.05). Statistical study power was calculated for both study groups based on weight, LV longitudinal strain, and LA minimal volume index using statistical tests. Study power was 0.99 in a total sample size of 72 subjects, with an alpha level of 0.05. 

Correlation analysis was performed using the Spearman correlation.

To evaluate the independent influence of obesity, BP, HR, and lipids on echocardiographic parameters, stepwise multiple linear regression analysis was performed using LV longitudinal, circumferential, and radial strain, as well as RV longitudinal strain, as dependent variables. Statistical tests were two-sided, and a *p*-value of <0.05 was considered statistically significant.

## 3. Results

### 3.1. Population

The main anthropometric, clinical, and lipid characteristics of the groups are shown in Table 1. The groups were matched for age and sex. Obese children had a significantly higher BMI, BMI-z score, BSA, SBP, and DBP (*p* < 0.001) as well as heart rate (*p* = 0.003), compared with control children. None of the obese children had a diagnosis of arterial hypertension, as their blood pressure (SBP and DBP) did not exceed the 90‰ BP percentiles according to age, sex, and height [14]. The level of total cholesterol was also significantly greater in the obese group (*p* < 0.001).

### 3.2. Conventional and Doppler Echocardiographic Characteristics

The conventional echocardiographic data are shown in Table 2. Obese children were characterized by a thicker LV posterior wall, enlarged LV mass and their z-scores (*p* < 0.001), and enlarged LV end-diastolic volume (*p* = 0.04), while LV volume (end-diastolic and end-systolic) z-scores did not differ significantly between the groups. Higher values of indexed LV mass were found in obese children (*p* < 0.001).

Tissue Doppler echocardiography revealed an impairment of LV and RV systolic and diastolic function (significantly decreased peak systolic velocity of the septal mitral annulus (Sm) and the tricuspid annulus (St) (*p* = 0.024, *p* = 0.004); significantly decreased early (Em) and late peak diastolic velocity (Am) of the septal mitral annulus (*p* = 0.013, *p* < 0.001); significantly decreased late peak diastolic velocity (At) of the tricuspid annulus (*p* = 0.004)) in obese children. LV and RV early and late filling velocity, LV filling pressure (E/Em) was not different between groups. 

### 3.3. Ventricular 2D Speckle Tracking Analysis

**Figure 1:**
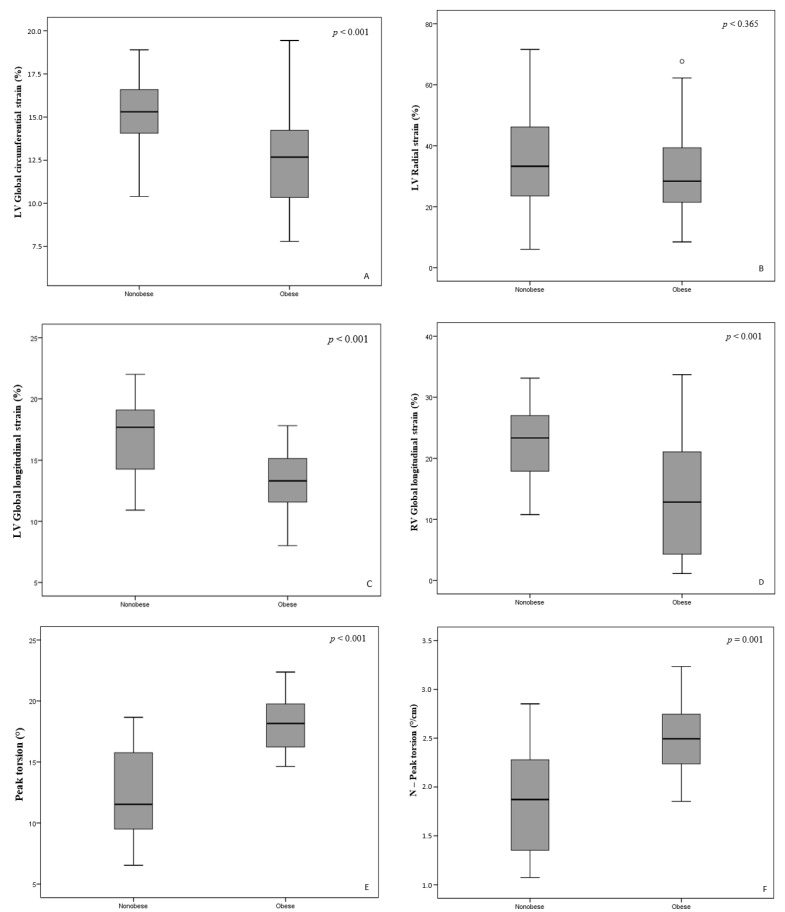
Comparison of the circumferential, radial strain of left ventricle (**A**,**B**) and longitudinal strain of left and right ventricle) (**C**,**D**), torsion, and normalized torsion of left ventricle (**E**,**F**) between obese and nonobese children.

Ventricular deformation parameters are shown in Figure 1. LV and RV global longitudinal strain was significantly reduced in obese children (−13.3% ± 2.88% vs. −16.87% ± 3.39% and −12.51% ± 10.09% vs. −21.51% ± 7.42%, *p* < 0.001). LV global circumferential strain (−17.0% ± 2.7% vs. −19.5% ± 2.9%, *p* < 0.001) was also significantly reduced in obese children. There was no significant difference in LV global radial strain between the groups (31.36% ± 14.88% vs. 34.81% ± 16.02%, *p* = 0.365). LV rotational parameters—torsion and normalized torsion—were significantly greater in obese than nonobese children (17.94° ± 2.07° vs. 12.45° ± 3.94°, *p* < 0.001 and 2.49° ± 0.4°/cm vs. 1.86° ± 0.61°/cm, *p* = 0.001, respectively). Significant inverse correlations were found between global LV and RV longitudinal strain and BMI (*r* = −0.526, *p* < 0.01 and *r* = −0.434, *p* < 0.01, respectively), as well as between global LV and RV longitudinal strain and hypercholesterolemia (*r* = −0.417, *p* < 0.01 and *r* = −0.274, *p* < 0.01, respectively). 

### 3.4. Atrial Volumetric Data and 2D Strain Analysis

The volumetric LA and RA data, the values of LA and RA strain are shown in Table 3. Only LA peak negative strain was significantly decreased in obese children (*p* < 0.01). LA peak positive, total strain, RA peak negative and peak positive, and total strain did not differ between the obese and nonobese children. Volumetric analysis showed significantly increased minimal LAVi and RAVi (the volume indexes during atrial contraction) (*p* < 0.001) as well as maximal LAVi and RAVi (the volume indexes at the end of systole) (*p* < 0.001) in obese children. RA reservoir volume, indexed to BSA, was higher in obese children, while LA reservoir volume, indexed to BSA, did not differ between groups. Interestingly, significantly higher values of LA and RA reservoir volume indexes, also indexed to their height raised to the power of 2.7, were found in obese children (*p* < 0.001). LA and RA stroke volume was significantly increased in obese children (*p* = 0.02 and *p* < 0.001, respectively). Although the total emptying fraction did not differ between groups, it tended to be smaller in the obese group.

Significant correlations were found between LA and RA reservoir volume and BMI (*r* = 0.506, *p* = 0.001 and *r* = 0.562, *p* = 0.001, respectively) as well as between LA reservoir volume and LV end-diastolic volume (*r* = 0.255, *p* = 0.005). A significant inverse correlation was observed between LA peak negative strain and BMI (*r* = −0.407, *p* = 0.001) as well as between LA peak negative strain and total cholesterol (*r* = −0.251, *p* = 0.04). No significant correlations between LA peak negative strain and LA volumes or indexes, LV parameters obtained by conventional or tissue Doppler echocardiography were observed.

### 3.5. Multivariate Stepwise Linear Regression Analysis

Stepwise multiple regression analysis showed that LV global circumferential, radial, longitudinal strain and RV global longitudinal strain were independently associated with the BMI-z score. There was no relationship between ventricular strain parameters and LDLP, HR, SBP, and DBP indexed by the z-score (Table 4).

### 3.6. Reproducibility

Intraobserver agreement as assessed by ICC was 0.1 (95% CI, −0.7 to 0.9) for LV longitudinal strain and −0.1 (95% CI, −0.91 to 0.7) for atrial peak negative strain.

## 4. Discussion

The data of our study provides additional support that childhood obesity is independently associated with changes in cardiac structure and function. The results show that obese children without comorbidities had a subclinical impaired atrial and ventricular systolic and diastolic function at a relatively early age (the age of the study group was 13.51 ± 2.15 years).

Using conventional echocardiography, our findings were that obese children had a larger LV mass and LV posterior wall thickness, increased LV end-diastolic volume, increased LA and RA volume. Obesity is related to insulin resistance, water and salt retention, which leads to higher total blood volume and increased preload [15,16,17]. Increased preload predisposes the body to LA and LV dilatation, LV remodeling and LV hypertrophy [18].

In line with other studies, our findings showed that obese children had increased heart rates and cholesterol levels compared to controls [17,19]. Increased activity of the sympathetic nervous system in obese children leads to increased afterload, which can result in LV hypertrophy.

On the other hand, dyslipidemia, as a part of metabolic syndrome, is a metabolic factor influencing vascular changes [1]. In our study, the cholesterol level was significantly higher in the obese group. Increased systemic vascular resistance leads to increased cardiac work and ventricular hypertrophy [18]. 

Assessing the systolic function of LV and RV by conventional echocardiography, the global LV ejection fraction (LVEF), tricuspid annulus motion amplitude did not differ between obese and nonobese children. Assessing the limitations of the conventional echocardiography measurement of LV and RV function [20], age-dependent normal ranges of the 2D strain of the LV and RV were reviewed in healthy children, using 2D speckle tracking echocardiography technology [21,22,23].

According to the data obtained by 2D speckle tracking echocardiography, LV and RV global longitudinal, as well as LV global circumferential, strains were significantly decreased in obese children. We found lower strain values in obese children using this technology, compared to the works by other authors [20,22,24,25]. In our opinion, in obese children with a relatively enlarged ventricle, the curvature of the ventricular free wall is relatively increased, which might contribute to the lower strain values observed. 

The results of our study are consistent with the findings of previous studies that analyzed LV and RV systolic long-axis function. Longitudinal strain assessed in obese children and young adults without high blood pressure was reduced, compared with healthy children [24,25,26,27,28]. In the present study, only 2D strain parameters revealed a subclinical reduction of LV and RV systolic function in obese children. 

Changes in longitudinal strain may be observed due to affecting the subendocardial layer [27]. The reasons are likely to be related to myocardial structure abnormality in obesity. Irregular adipose tissue distribution between myocardial cells and increased pressure lead to myocardial cell atrophy and cardiac dysfunction [28,29]. 

In the present study, only radial strain values, derived from 2D speckle tracking echocardiography, were comparable in obese and nonobese children. 

The proposed mechanism for this was described by Saltijeral et al. as a compensation mechanism to maintain contractility at an early stage of obesity cardiomyopathy [5].

This explains that “normal” LVEF was not equal to normal systolic function, as compensatory mechanisms in different strain directions can preserve global EF. In the early stages of heart disease, there is a reduction in the longitudinal myocardium contraction; in the later stages, reduction in the circumferential and radial contractions occurs and leads to a reduced LVEF [5,30]. 

LV morphological alterations observed in our study have shown that LV remodeling determines functional LV impairment. Decreased global circumferential strain, as well as increased apical rotation and torsion, were indicators of impaired diastolic function in obese children. LV torsion is after-load dependent, and a chronic increase in afterload leads to increasing apical rotation and LV torsion [13,16]. Interestingly, increased torsion has been reported in asymptomatic type 1 diabetic patients, and in patients with early-stage diastolic dysfunction [13]. To our knowledge, there are no data about rotational parameters in obese children. 

Despite weak correlations between LV and RV global longitudinal strain and BMI, our study showed that BMI was independently related to LV and RV longitudinal, LV circumferential, and LV radial strains. These results are consistent with the data of previous studies, where BMI was an independent predictor of worsening LV and RV systolic and diastolic function [3,5,24]. 

Since atrial function is influenced by ventricular geometry and function, we evaluated atrial function using conventional and 2D speckle tracking echocardiography. In our study, we expected an increase in atrial strain as a result of volume overload, but conversely, we found a significant reduction of LA strain in contraction among obese children. This means that contractile atrial function is impaired in obese children. When LA enlargement exists, it would be clinically helpful to have parameters that could distinguish normal from impaired LA contractile function. LA contractile function is dependent on preload stretch and afterload, represented by LV end-diastolic pressure [28]. 

However, in our study, LA and RA strain in relaxation (peak positive strain, reflecting atrial conduit function) did not differ between groups. These findings contradict the results of a study by di Salvo et al. [24] who reported significantly decreased LA relaxation strain in obese children. Wakami et al. [31] found a significant relation between LA strain in relaxation and LV hemodynamic measurements. Inverse correlations were found between LA strain in relaxation and LV end-diastolic pressure, and LV end-systolic volume, while the correlation between LA strain in relaxation and LV ejection fraction was positive. As LV end-systolic volume, LV ejection fraction, LV early and late filling velocity, and LV early filling deceleration time did not differ between the groups, it is plausible that LA strain in relaxation was not changed in the obese children in our study.

The results of our study suggest a weak association between early myocardial dysfunction in obese children. These findings suggest turning clinicians’ attention to the importance of obesity treatment and weight loss in childhood as early as possible. 2D speckle tracking echocardiography could help assess the potential reversibility of myocardial dysfunction during obesity treatment with a return to normal weight.

The most important limitation of the study was the small cohort. Another limitation of our study was an incomplete metabolic profile (fasting glucose, fasting insulin, HOMA IR) in both obese and nonobese children, as a metabolic abnormality exerts an independent effect on cardiac function. Physical activity was measured merely by a survey of patients and their parents, without using any validated instrument. Data on the renin-angiotensin-aldosterone system and the sympathetic nervous system were not investigated. Additionally, the study design does not allow providing information about the potential reversibility of the changes with weight loss over time. Larger-scale, long-term studies are necessary to confirm the clinical importance of these study results.

## 5. Conclusions

2D speckle tracking echocardiography is beneficial for the early detection of regional LV systolic and diastolic dysfunction with preserved ejection fraction, as well as additional RV and atrial involvement in obese children. Obesity (BMI above the 95th percentile) may negatively influence atrial and ventricular function, measured by 2D speckle tracking echocardiography. Our results support that obese children, despite their being apparently healthy, may have subclinical myocardial dysfunction.

## Figures and Tables

**Table 1:** Clinical characteristics.

Variable	Obese *n* = 35	Nonobese *n* = 37	*p* Value
Gender, boys/girls, *n*	20/15	18/19	0.490
Age, years	13.51 (2.1)	13.43 (1.7)	0.945
Heart rate, bpm	86.43 (10.73)	78.12 (12.28)	0.003
Systolic blood pressure, mmHg	126.14 (13.76)	113.22 (7.22)	<0.001
Diastolic blood pressure, mmHg	78.74 (8.59)	70.70 (6.71)	<0.001
Height, median (IQR), cm	168 (138–178)	172.61 (140–191)	0.015
Weight, kg	91.06 (18.65)	56.11 (9.91)	<0.001
Body mass index, kg/m^2^	33.12 (5.15)	19.08 (3.08)	<0.001
Body mass index, z-score	0.88 (0.63)	−0.83 (0.38)	<0.001
Body surface area, m^2^	2.01 (0.26)	1.64 (0.17)	<0.001
Total cholesterol, mmol/L	4.46 (1.03)	3.34 (0.71)	<0.001
LDLP, mmol/L	2.74 (1.1)	2.14 (0.58)	0.060
HDLP, median (IQR), mmol/L	1.09 (0.72–3.21)	1.14 (1–1.65)	0.045
Triglycerides, median (IQR), mmol/L	1.26 (0.50–3.16)	1.24 (0.85–1.84)	0.502

Values are mean (standard deviation) unless otherwise indicated. IQR, interquartile range, LDLP, low-density lipoprotein; HDLP, high-density lipoprotein.

**Table 2:** Conventional and Doppler echocardiographic characteristics.

Parameters	Obese *n* = 35	Nonobese *n* = 37	*p* Value
LV end-diastolic volume, mL	100.54 (21.42)	85.05 (22.30)	0.04
LV end-systolic volume, mL	24.94 (8.23)	24.68 (12.45)	0.915
Interventricular septum, mm	8.71 (1.34)	8.24 (1.21)	0.158
LV posterior wall, mm	8.83 (1.29)	7.70 (1.31)	<0.001
LV ejection fraction, %	69.29 (6.98)	67.32 (8.97)	0.243
LV fractional shortening, %	43.71 (4.71)	45.35 (5.66)	0.188
LV mass, g	115.81 (32.22)	80.12 (18.31)	<0.001
LV mass/height ^2.7^, g/m ^2.7^	30.35 (6.12)	19.0 (3.93)	<0.001
LV end-diastolic volume z-score	0.13 (0.98)	−0.12 (1.02)	0.291
LV end-systolic volume z-score	0.01 (0.78)	−0.01 (1.18)	0.915
Interventricular septum z-score	0.18 (1.04)	−0.18 (0.94)	0.122
LV posterior wall z-score	0.41 (0.92)	−0.39 (0.93)	<0.001
LV ejection fraction z-score	0.12 (0.86)	−0.12 (1.11)	0.306
LV mass z-score	0.15 (0.94)	−0.13 (0.97)	0.012
LV early filling velocity, m/s	0.88 (0.18)	0.86 (0.09)	0.609
LV late filling velocity, m/s	0.59 (0.15)	0.55 (0.09)	0.284
E/Am	1.52 (0.40)	1.59 (0.38)	0.459
LV early filling deceleration time, median (IQR), m/s ms	147 (24–217)	152 (120–230)	0.238
Sm, median (IQR), cm/s	0.092 (0.04–0.4)	0.11 (0.06–0.19)	0.024
Em, median (IQR), cm/s	0.16 (0.07–0.24)	0.19 (0.08–0.24)	0.013
Am, median (IQR), cm/s	0.06 (0.04–0.11)	0.16 (0.06–0.16)	<0.001
E/Em, median (IQR)	7 (4.1–16.4)	7.5 (3–14.6)	0.451
RV basal diameter, mm	29.1 (3.20)	28.3 (2.32)	0.843
RV early filling velocity, m/s	1.08 (14.65)	1.06 (20.24)	0.172
RV late filling velocity, m/s	0.61 (0.12)	0.58 (0.11)	0.295
Tricuspid annulus motion amplitude, mm	20.44 (0.42)	18.53 (0.81)	0.752
St, median (IQR), cm/s	0.76 (0.041–0.9)	0.09 (0.05–0.15)	0.004
Et, median (IQR), cm/s	0.13 (0.09–0.22)	0.13 (0.06–0.19)	0.544
At, median (IQR), cm/s	0.06 (0.04–0.11)	0.08 (0.06–0.16)	0.004

Values are mean (standard deviation) unless otherwise indicated. LV, left ventricle; E/A, ratio of early and late LV filling velocity; Sm, peak systolic velocity of the septal mitral annulus; Em, early peak diastolic velocity of the septal mitral annulus; Am, late peak diastolic velocity of the septal mitral annulus; E/Em, left ventricle filling pressure; St, peak systolic velocity of the tricuspid annulus; Et, early peak diastolic velocity of the tricuspid annulus; At, late peak diastolic velocity of the tricuspid annulus; RV, right ventricle; IQR, interquartile range.

**Table 3:** Atrial longitudinal strain and volumetric data.

Parameter	Obese *n* = 35	Nonobese *n* = 37	*p* Value
LA peak negative strain, %	−2.05 (2.33)	−4.87 (2.97)	<0.001
LA peak positive strain, %	29.98 (10.36)	27.88 (12.62)	0.297
Total LA strain, %	31.88 (12.49)	28.97 (15.18)	0.126
LAVi max, mL/m^2^	29.55 (17.01)	24.66 (10.75)	<0.001
LAVi min, mL/m^2^	9.94 (3.76)	6.66 (1.94)	<0.001
Total LA stroke volume, mL	18.93 (10.38)	15.03 (5.86)	0.02
LARVi, mL/m^2^	28.72 (8.50)	32.81 (7.65)	0.348
LARVi/ height ^2.7^, mL/m ^2.7^	9.79 (3.52)	6.36 (1.82)	<0.001
Total LA emptying fraction, %	47.16 (19.42)	54.14 (12.65)	0.286
RA peak negative strain, %	−4.79 (6.60)	−4.88 (2.45)	0.818
RA peak positive strain, %	36.05 (26.22)	34.77 (20.29)	0.927
Total RA strain, %	32.99 (26.83)	31.88 (22.21)	0.219
RAVi max, mL/m^2^	19.54 (6.50)	15.83 (5.77)	0.011
RAVi min, mL/m^2^	11.06 (3.85)	7.50 (2.56)	0.001
Total RA stroke volume, mL	17.78 (10.19)	11.94 (6.43)	0.01
RARVi, mL/m^2^	29.66 (8.50)	24.44 (7.65)	0.01
RARVi/ height ^2.7^	10.15 (3.16)	6.07 (2.14)	<0.001
Total RA emptying fraction, %	43.96 (15.53)	46.10 (12.26)	0.374

Values are mean (standard deviation). LA, left atrium; RA, right atrium; LAVi, left atrial volume index; RAVi, right atrial volume index; LARVi, left atrial reservoir volume index; RARVi, right atrial reservoir volume index.

**Table 4:** Multivariate stepwise linear regression analysis.

Parameters	LV Circumferential Strain		LV Radial Strain		LV Longitudinal Strain		RV Longitudinal Strain	
	β (95% CI)	*p*	β (95% CI)	*p*	β (95% CI)	*p*	β (95% CI)	*p*
BMI z-score	−1.548 (−2.75; −0.35)	0.012	−6.817 (−11.43; −2.21)	0.004	−1.353 (−2.44; −0.27)	0.016	−4.576 (−7.02; −2.13)	0.001
HR z-score	0.240 (−0.78; 1.26)	0.640	1.205 (−2.80; 5.21)	0.550	0.219 (−0.75; 1.19)	0.653	0.536 (−1.64; 2.71)	0.624
SBP z-score	0.214 (−1.00; 1.43)	0.727	0.609 (−4.02; 5.24)	0.793	−0.139 (−1.27; 0.99)	0.807	−0.197 (−2.69; 2.29)	0.875
DBP z-score	−0.968 (−2.25; 0.32)	0.137	2.464 (−2.40; 7.33)	0.315	−0.661 (−1.83; 0.51)	0.264	0.678 (−2.01; 3.36)	0.615
LDLP z-score	−0.044 (−1.04; 0.95)	0.930	3.905 (−0.314; 8.13)	0.069	−0.978 (−1.97; 0.02)	0.055	−1.585 (−3.79; 0.63)	0.157

LV, left ventricle; RV, right ventricle; BMI, body mass index; HR, heart rate; SBP, systolic blood pressure; DBP, diastolic blood pressure; LDLP, low-density lipoprotein.

## Data Availability

Not applicable.
